# Letter to the editor regarding: ‘association between triglyceride-glucose index upon admission and the subsequent occurrence of acute kidney injury in adult patients with diabetic ketoacidosis: a single center retrospective cohort study’

**DOI:** 10.1080/07853890.2025.2604878

**Published:** 2025-12-26

**Authors:** Yi-Fan Guo, Mao-Sheng Xu

**Affiliations:** Department of Radiology, the First Affiliated Hospital of Zhejiang Chinese Medical University (Zhejiang Provincial Hospital of Traditional Chinese Medicine), Hangzhou, China

To the editor

We read with great interest the retrospective cohort study by Jin et al. assessing the association between the triglyceride–glucose (TyG) index at admission and acute kidney injury (AKI) in adults with diabetic ketoacidosis (DKA) [1]. The authors reported a nonlinear relationship between TyG and subsequent AKI and proposed a threshold around 10.92 as a practical cut-off for risk stratification. This is an important and clinically relevant topic, given the high burden of renal complications in DKA. However, we would like to raise several methodological concerns that may limit the robustness and generalisability of their conclusions.

First, the derivation and use of the TyG cut-off at 10.92 appear to be strongly data driven. In this single cohort, TyG was divided into quartiles, with the lower bound of the highest quartile coinciding with 10.92, and restricted cubic spline analyses suggested a similar turning point. The same value was then adopted as a clinical cut-off and used as the exposure in propensity score matching. A threshold determined entirely within one dataset is prone to overfitting and optimism bias, so the observed risk gradient may be larger than would be seen in routine clinical practice. Without external validation, the ‘optimal’ cut-off is likely to shift across populations, and building algorithms or management bundles around this value risks conveying a level of validation that has not yet been achieved [2].

Second, the reporting of AKI definition, baseline renal function and confounder control seems incomplete. Because urine output criteria were unavailable, AKI was defined solely by serum creatinine, yet the manuscript does not clearly specify how baseline creatinine was obtained (previous outpatient measurement, admission value or a back-calculated estimate), even though this choice directly affects AKI incidence and KDIGO staging [3]. Moreover, the distribution of AKI stages is not presented, so it is unclear whether TyG is particularly associated with moderate-to-severe AKI. In the multivariable models, baseline kidney function, indices of DKA severity (such as pH, bicarbonate or anion gap) and key treatment-related factors appear to be only partially accounted for, leaving room for substantial residual confounding [4].

We therefore encourage the authors to provide a clearer description of baseline creatinine ascertainment, present AKI stage distribution and extend their models to incorporate baseline renal function, DKA severity indices and relevant treatment factors. Separate models aimed at causal interpretation and at risk prediction could help clarify whether TyG should be viewed primarily as a potential causal factor or as a low-cost risk marker. Ideally, the proposed TyG threshold should also be examined in an independent cohort, with appropriate internal or external validation [5].

In conclusion, Jin et al. contribute valuable data suggesting a nonlinear association between admission TyG and AKI in adults with DKA and highlighting a possible high-risk range. With more transparent definitions, more comprehensive adjustment for confounding and independent validation of the data-derived cut-off, this line of research may provide a stronger basis for renal risk stratification and early management in this vulnerable population.

## Data Availability

Data availability is not applicable to this article as no new data were created or analyzed in this study.
